# Association between temporalis muscle thickness and functional outcomes in acute stroke: A meta-analysis and GRADE approach

**DOI:** 10.1016/j.jnha.2025.100614

**Published:** 2025-06-21

**Authors:** Yao-Chung Yang, Chun-Hao Yin, Pei-Chin Lin, Yow-Ling Shiue

**Affiliations:** a Department of Biological Sciences, National Sun Yat-Sen University, Kaohsiung, Taiwan; b Division of Neurosurgery, Department of Surgery, Kaohsiung Veterans General Hospital, Kaohsiung, Taiwan; c Department of Medical Education and Research, Kaohsiung Veterans General Hospital, Kaohsiung, Taiwan; d Department of Pharmacy, Kaohsiung Veterans General Hospital, Kaohsiung, Taiwan; e Department of Pharmacy, School of Pharmacy, Kaohsiung Medical University, Kaohsiung, Taiwan; f Department of Biological Sciences, National Sun-Yat-sen University, Kaohsiung, Taiwan; g Institute of Biomedical Sciences, National Sun Yat-sen University, Kaohsiung, Taiwan

**Keywords:** Sarcopenia, Temporalis muscle, Stroke, Ischemia, Prognosis, Dysphagia

## Abstract

**Background:**

Sarcopenia is associated with poor prognosis in patients with acute stroke. While temporalis muscle thickness (TMT) and area (TMA) have been studied in various conditions, their association with stroke prognosis remains unclear.

**Methods:**

To investigate the validity of TMT and TMA as poststroke outcome predictors, we performed a comprehensive search of the PubMed, CENTRAL, and Embase databases to identify pertinent studies published up to October 31, 2024. A meta-analysis of the pooled estimates of the temporalis muscle predictors was conducted, and the evidence quality was assessed using the Grading of Recommendations Assessment, Development, and Evaluation (GRADE) approach.

**Results:**

This meta-analysis included 15 studies and revealed that both TMT and TMA were associated with functional outcomes and dysphagia risk in patients with stroke. Specifically, patients with better functional outcome had higher TMT value (mean difference [MD] = 0.84 mm, 95% confidence interval [CI] = 0.55–1.13, *I*^2^ = 45%). Likewise, patients with better functional outcomes had larger TMA values (MD = 65.99 mm^2^, 95% CI = 0.41–1.06, *I*^2^ = 85%). A lower TMT value at stroke onset was associated with increased dysphagia risk (MD = 1.63 mm, 95% CI = 0.74–2.52, *I*^2^ = 50%). Subgroup analyses showed that the association between TMT and functional outcome was more evident in individuals aged 50 years or older and in Asian populations, with no significant differences observed between sexes. The certainty of evidence according to GRADE assessment ranged from low to moderate.

**Conclusions:**

TMT and TMA measurements obtained from routine neuroimaging may serve as useful reference markers for functional prognosis in stroke patients.

## 1 Introduction

Stroke is the second and third leading cause of death and disability worldwide, respectively, accounting for 6.6 million deaths and 143 million disability-adjusted life years (DALYs) lost globally in 2019 [1]. Ischemic stroke accounts for >80% of cases, whereas hemorrhagic stroke is more disabling and more often fatal [2,3]. Over 70% of patients with stroke suffer long-term disability, with increasing age associated with unfavorable outcomes [4]. For patient management and rehabilitation planning, various validated predictors include clinical scales (National Institutes of Health Stroke Scale [NIHSS], prestroke-modified Rankin Scale [mRS]), imaging factors (lesion volume) [5], and composite scores, such as the ASTRAL score (Age, Severity of stroke measured by admission NIHSS, stroke onset to admission Time, Range of visual fields, Acute glucose, and Level of consciousness) [6] and the DRAGON score (Dense cerebral artery sign/early infarct signs, prestroke mRS, Age, Glucose level, Onset-to-treatment time, and NIHSS) [7].

In recent years, sarcopenia has been increasingly studied for its potential association with stroke outcomes. It is characterized by a progressive decline in muscle mass and strength and is associated with adverse stroke outcomes [[8], [9], [10], [11], [12]]. In stroke patients, sarcopenia is correlated with higher mortality, poorer functional outcomes, neurological deterioration, and higher aspiration pneumonia risk during hospitalization [[13], [14], [15]]. Early identification of sarcopenia based on diagnostic criteria is crucial for outcome prediction and rehabilitation planning [16,17]. Despite its potential clinical value, routine abdominal imaging to estimate skeletal muscle mass at the third lumbar vertebra (L3) level is not commonly performed in stroke patients with suspected sarcopenia [17]. Temporalis muscle thickness (TMT) measurement on routine cranial imaging (CT or MRI) may serve as a practical approach for muscle mass assessment and sarcopenia identification in patients with stroke [18,19]. Moreover, previous morphomic studies have demonstrated that TMT correlates with skeletal muscle cross-sectional area at the L3 level [20] and with total psoas muscle area [21], supporting its validity as a surrogate for global muscle mass. TMT is strongly correlated with muscle circumference and moderately with grip strength, suggesting its possible utility as a marker of nutritional status and sarcopenia [22].

TMT’s predictive value has been demonstrated in brain conditions, including glioblastoma, as an independent prognostic marker [23]. Additional research has reported that both TMT and temporalis muscle area (TMA) are independently associated with sarcopenia-related clinical outcomes, including neurological status and long-term functional recovery in subarachnoid hemorrhage patients [24]. However, findings are not universal; a recent study in a Central European cohort reported that neither initial TMT nor TMA significantly predicted neurological outcomes in aneurysmal subarachnoid hemorrhage [25]. These inconsistencies underscore the need for a systematic synthesis of available evidence to clarify the prognostic value of TMT and TMA in stroke populations.

Clinically, various methods exist for predicting stroke outcomes; however, in emergency settings, collecting comprehensive data can be challenging due to factors such as patients’ altered consciousness or the unavailability of certain clinical parameters. This limitation highlights the need for developing alternative, accessible prognostic predictors. As TMT measurement can be easily obtained from routine cranial imaging, it has been proposed as a potential candidate for outcome prediction. Although the literature defines sarcopenia or low muscle mass based on mean TMT values below a certain threshold, there is currently no universally accepted cutoff. Moreover, most studies on TMT and stroke prognosis are retrospective and geographically limited. Therefore, a comprehensive meta-analysis is warranted to provide substantial evidence for future guidelines and clarify TMT’s role as a prognostic indicator in patients with stroke.

## 2 Methods

### 2.1 Data sources and search strategy

This systematic review and meta-analysis followed PRISMA guidelines [26] and was registered in PROSPERO (CRD42024558812). We searched PubMed, Cochrane (CENTRAL), and Embase databases until October 31, 2024, using keywords, MeSH, and Emtree terms including “stroke,” “subarachnoid hemorrhage (SAH),” “intracerebral hemorrhage (ICH),” “intracranial hemorrhage,” “temporalis muscle,” and “temporal muscle” (Table S1). We searched ClinicalTrials.gov using the keywords “acute stroke” and “temporal muscle” to identify ongoing or unpublished studies. We included studies in any language and manually reviewed the reference lists for additional relevant studies.

### 2.2 Eligibility criteria

Two investigators (YC Yang & CH Yin) independently screened the studies, with disagreements resolved by a third reviewer (PC Lin). The inclusion criteria were as follows: (1) adult patients with ischemic or nontraumatic hemorrhagic stroke (SAH and ICH); (2) diagnosis confirmed by CT or MRI; (3) cases in which TMT was used to predict poor functional outcome or sequela; and (4) randomized controlled trials or observational studies. We excluded studies on traumatic brain injury, pediatric cases, and abstract-only publications.

### 2.3 Data extraction and study quality

Two authors (YC Yang & CH Yin) independently extracted data: publication year, country, study design, patient number, demographics, stroke etiology, study period, temporalis muscle measurement details (equipment, methodology, unit), sarcopenia cutoff values, median follow-up duration, and outcomes. In cases where multiple publications originated from the same dataset, the most comprehensive, relevant, or most recently published study was included to avoid duplicate data extraction. We considered TMT and temporalis muscle area (TMA) measurements, extracting odds ratios (OR), 95% confidence intervals (CIs), and cutoff values for poor outcomes. For studies reporting only case numbers and muscle values, we recorded continuous data. Missing data were requested from original authors. Study quality was assessed using the Newcastle–Ottawa scale for nonrandomized studies based on selection (four items), comparability (one item), and exposure/outcome (three items) [27]. Scores ≥7 indicate high quality, 4–6 medium quality, and ≤4 low quality.

### 2.4 Statistical analysis

First, we summarized the main results of the included studies, according to the conclusions of the original authors, and constructed a comprehensive results matrix for temporalis muscle measurement. Second, if an outcome concerning the specific muscle measurement unit predictors was reported in at least two studies, we conducted a meta-analysis on those predictors. The effect size of temporalis muscle measurement predictors of poor outcome was presented as the pooled odds ratio. If the temporalis muscle measurements were reported only as continuous data, we calculated the effect size as the mean difference (MD). If these data were skewedly distributed and thus presented as medians and interquartile ranges, we employed the method described by Wan et al. [28] to convert them to means and standard deviations. A random-effects model was conservatively used for statistical analyses due to the possibility of medical management differences. The equivalent *z*-test was conducted, and in the case of *P* <  0.05, the pooled result was considered statistically significant. We used Cochran’s *Q* test (*P* <  0.10) and the *I*^2^ statistic to evaluate the heterogeneity between studies. Substantial statistical heterogeneity between studies was defined by *I*^2^ values of > 50%, and values of > 75% indicated considerable heterogeneity as per the *Cochrane Handbook for Systematic Reviews of Interventions* [29,30]. We also performed prespecified subgroup analyses comparing the following factors: age (<50, 50–64, 65–79, and ≥80 years) [31], sex (female vs. male), ethnicity (Asian vs. European), imaging modality (CT vs. MRI), and stroke type (Ischemic stroke vs. Hemorrhagic stroke). A meta-analysis was performed using Review Manager (RevMan) 5.4.1 (The Cochrane Collaboration, 2020) and forest plots were presented.

### 2.5 GRADE the evidence

We used the Grading of Recommendations, Assessment, Development, and Evaluations (GRADE) system to evaluate the quality of evidence for each outcome [32], starting with low confidence for observational studies. Confidence levels were adjusted based on risk of bias, inconsistency, indirectness, and imprecision [[33], [34], [35]]. Enhancing factors included large effect size, dose–response relationship, and plausible residual confounding. The final recommendations were based on the effect estimate confidence and relevant factors [36].

## 3 Results

### 3.1 Study selection and characteristics of included studies

**Fig. 1:**
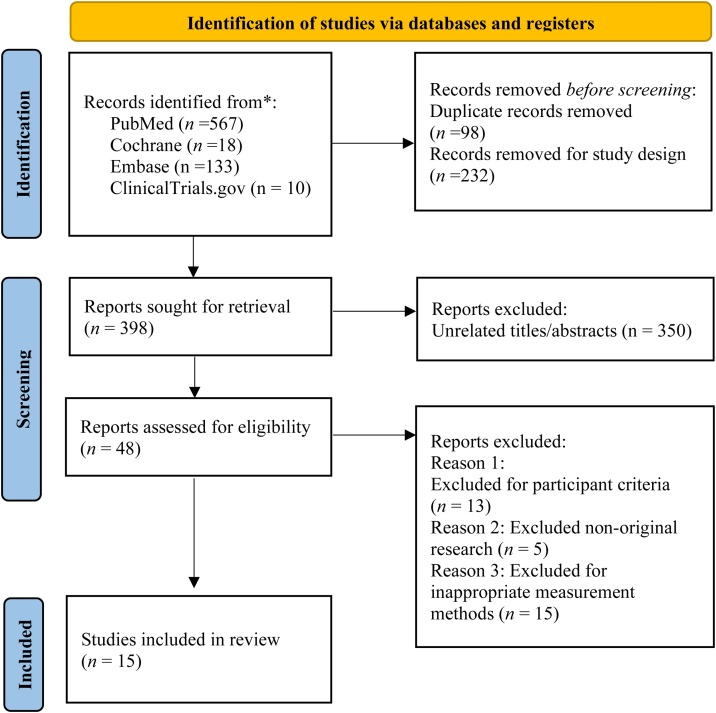
PRISMA flowchart summarizing study identification and selection.

A total of 728 records were identified through database and register searches, including PubMed (*n* = 567), Embase (*n* = 133), Cochrane Library (*n* = 18), and ClinicalTrials.gov (*n* = 10). After removing 98 duplicates and 232 records excluded based on study design or type, 398 records remained. At this stage, 350 were excluded after screening titles/abstracts (unrelated abstracts, pediatric studies, and traumatic brain injury cases). Of the remaining 48 papers, 33 were excluded after full-text review (nonoriginal research, inappropriate participant criteria, and inappropriate measurement methods). The PRISMA flow diagram is shown in Fig. 1. The final 15 included studies were published between 2020 and 2024 and included 51–657 patients [9,25,[37], [38], [39], [40], [41], [42], [43], [44], [45], [46], [47], [48], [49]]. Of these, nine studies examined acute ischemic stroke [37,40,[42], [43], [44],^46^,[38], [39], [40], [41], [42], [43], [44], [45], [46], [47], [48], [49]], five focused on aneurysmal subarachnoid hemorrhage (aSAH) [25,38,39,41,45], and one study included both acute ischemic stroke and intracerebral hemorrhage patients [9]. The mean age of the participants was 56–79 years, and 37%–80.4% were female. TMT was assessed using CT in 11 studies, of which four also included TMA measurements, all obtained via CT [9,25,[37], [38], [39],^41^,^42^,^45^,^47^,[38], [39], [40], [41], [42], [43], [44], [45], [46], [47], [48], [49]]. For TMT measurements, studies used one of two approaches: either measuring on a plane that was 5 mm above the orbital roof or at the level of the orbital roof. In both cases, the thickness was measured at the level of the Sylvian fissure. All four studies that reported the TMA performed measurements on a plane that was 5 mm above the orbital roof [25,38,39,45]. The included studies reported various outcomes, with follow-up periods mostly exceeding three months. mRS was the most common outcome (9/15), followed by dysphagia (2/15), survival time, mortality, Korean version of the Modified Barthel Index (K-MBI), and Montreal Cognitive Assessment (MoCA) scores (Table 1).

**Table 1:** Characteristics of 15 observational studies included in the analysis.

Study	Study design	Country	Patient (*n*)	Sex (male)	Age	Etiology	Study period	Measurement method	TM measuring position	Sarcopenia cutoff value^a^	Follow up (months)	Outcome
Dubinski D, 2023	retro	Germany	287	128 (45%)	71	Cerebellar AIS	8/2016 to 6/2021	CT/MRI; TMT	Plane with max TMT	TMT: 5.5mm	12	mRS score
Karadag C, 2023	retro	Germany	478	162 (34%)	56	aSAH	1/2015 to 10/2022	CT; TMT/TMA	5 mm above orbital roof	Mean TMT: 5.9 & 5.6 mm	6	mRS score
									Sylvian fissure	Mean TMA: 296 & 271 mm		
Katsuki M, 2020	retro	Japan	298	90 (30%)	63.7	aSAH treated by Coiling	1/2009 to 12/2019	CT; TMT/TMA	5 mm above orbital roof	TMT: 5 mm	6	mRS score
									Sylvian fissure	TM: 200 mm^2^		
Katsuki M, 2021	retro	Japan	127	45 (35%)	60.6	aSAH treated by clipping	1/2009 to 12/2019	CT; TMT/TMA	5 mm above orbital roof	TMT: 4.9 mm (female), 6.7 mm (male); TMA: 193 mm^2^ (female), 333 mm^2^ (male)	6	mRS score
									Sylvian fissure			
Li YX, 2022	retro cohort	China	265	166 (63%)	28−92	AIS	1/2015 to 12/2018	MRI; TMT	Orbital roof	–	–	Survival time
									Sylvian fissure			
Lim JX, 2022	retro	Singapore	51	10 (20%)	58	aSAH	1/2014 to 12/2015	CT; TMT	5 mm above orbital roof	TMT: 5.5 mm	12	mRS score
									Sylvian fissure			
Lin YH, 2023	retro	Taiwan	657	342 (52%)	72	AIS treated by EVT	11/2014 to 12/2021	CT; TMT	Orbital roof	Mean TMT: 6.81 & 6.05 mm	3	mRS score
									Sylvian fissure			
Namgung HG, 2023	retro & prosp	Korea	126	9 (45%)	79	AIS	1/2020 to 12/2022	MRI; TMT	Orbital roof	Mean TMT: 6.8 & 6.2 & 5.7 mm	3	MoCA scale
									Sylvian fissure			
Nozoe M, 2019	Cross-sectional	Japan	289	163 (56%)	76	AIS & ICH	8/2017 to 12/2019	CT; TMT	Orbital roof	TMT: 5.64 & 5.30 mm; TMT: 4.625 mm	3	mRS score
									Sylvian fissure			
Park J, 2023	retro	Korea	84	52 (62%)	69	AIS	11/2021 to 8/2022	MRI; TMT	Orbital roof	Mean TMT: 9.88 & 9.72 mm	1 week	K-MBI scores
									Sylvian fissure			
Rodrigues RS, 2022	prosp	Brazil	361	151 (42%)	57.1	aSAH	1/2018 to 12/2019	CT; TMT/TMA	5 mm above orbital roof	TMT: 6.25 mm	6	mRS score
									Sylvian fissure	TMA: 266.1 mm^2^		
Sakai K, 2021	retro	Japan	70	43 (61%)	75.6	AIS	4/2017 to 3/2018	MRI; TMT	Orbital roof	Median TMT: 5.75 & 4.7 mm	6	Dysphagia
									Sylvian fissure			
Tutal Gürsoy G, 2023	retro	Turkey	147	72 (49%)	67.6	AIS	1/2021 to 9/2022	CT; TMT	Orbital roof	TMT: 5.2 mm	3	Mortality
									Sylvian fissure			
Yang SM, 2023	retro	Taiwan	148	68 (46%)	73.1	AIS	12/2018 to 3/2022	CT; TMT	Orbital roof	TMT: 4.7 mm	12	Dysphagia
									Sylvian fissure			
Yang YC, 2024	retro	Taiwan	96	58 (60%)	69.5	AIS	8/2016 to 7/2022	CT; TMT	5 mm above orbital roof	Mean TMT: 6.66 & 5.32 mm	3	mRS score
									Sylvian fissure			

Abbreviation: TM = temporalis muscle; retro = retrospective study; AIS = acute ischemic stroke; CT = computed tomography; MRI = magnetic resonance imaging; TMT = temporalis muscle thickness; max = maximum; mRS = modified Rankin scale; aSAH = aneurysmal subarachnoid hemorrhage; TMA = temporalis muscle area; EVT = endovascular thrombectomy; prosp = prospective study; MoCA = Montreal cognitive assessment; ICH = intracranial hemorrhage; K-MBI = Korean version of modified Barthel index; RBC = red blood cell; CRP = C-reactive protein; mFisher = modified Fisher grade.

a Cutoff values are presented directly, while other values are labeled accordingly (e.g., mean, median).

### 3.2 Quality of included studies

All 15 studies were of high quality (Newcastle–Ottawa scores ≥ 7) [9,25,[37], [38], [39], [40], [41], [42], [43], [44], [45], [46], [47], [48], [49]]. Specifically, 3 studies did not provide comprehensive characteristics of their cohorts [41,44,46], and 2 studies did not adequately report the follow-up of cohorts (Table S2) [40,44]. Based on the GRADE approach, we evaluated the certainty of evidence for our key outcomes. Two clinically critical outcomes (the TMT and the TMA) were assessed in relation to functional outcomes (mRS). The evidence for both TMT and TMA was rated as moderate certainty. TMT’s dysphagia prediction showed low certainty. All three outcomes were downgraded due to serious imprecision but upgraded for the presence of a dose–response gradient. However, the certainty of evidence for TMT’s dysphagia prediction was further downgraded due to serious inconsistency, resulting in an overall low certainty rating. Considering that CT scans are routinely performed in stroke patients without additional radiation exposure, coupled with the timeliness of the examination and the clinically critical importance of the outcomes, we support a strong recommendation for the use of TMT and TMA as predictors in clinical practice. The results of our GRADE analysis are presented in Table 2.

**Table 2:** Grading of Recommendations Assessment, Development, and Evaluation (GRADE) approach evidence certainty and summary of findings of the clinical important outcomes.

			Certainty assessment					Certainty	Summary of findings		
Outcomes	No of studies	Study design	Risk of bias	Inconsistency	Indirectness	Imprecision	Other considerations	Overall certainty of evidence	Relative (95% CI)	Mean difference with temporal muscle measurement between good and poor functional outcomes (95% CI)	Importance
TMT and functional outcomes											
mRS	7	non-RCT	not serious	not serious	not serious	not serious	dose–response gradient	⨁⨁⨁◯ Moderate	–	MD **0.74 higher** (0.41 higher to 1.06 higher)	CRITICAL
Dysphagia	2	non-RCT	not serious	serious ^a^	not serious	not serious	dose–response gradient	⨁⨁◯◯ Low	–	MD **1.63 higher** (0.74 higher to 2.52 higher)	IMPORTANT
TMA and functional outcomes											
mRS	3	non-RCT	not serious	not serious	not serious	not serious	dose–response gradient	⨁⨁⨁◯ Moderate	–	MD **65.99 higher** (15.21 higher to 116.77 higher)	CRITICAL

The bold of the words in the columns of “summary of findings” means a statistical significance. *:⨁⨁⨁⨁ means the highest level of evidence certainty; if a ⨁ was replaced by a ◯, it means a downgrade of evidence certainty. There were four levels of certainty, high, moderate, low and very low, represented as ⨁⨁⨁⨁, ⨁⨁⨁◯, ⨁⨁◯◯ and ⨁◯◯◯, separately. Abbreviation: CI = Confidence interval; TMT = temporalis muscle thickness; mRS = modified Rankin Scale; RCT = randomized controlled trial; MD = Mean difference; TMA = temporalis muscle area. Explanations: a: substantial heterogeneity (*I*^2^ = 80%) and cannot find the source of heterogeneity.

### 3.3 Cross-country comparative matrix of TMT cutoff values and TMA findings

**Fig. 2:**
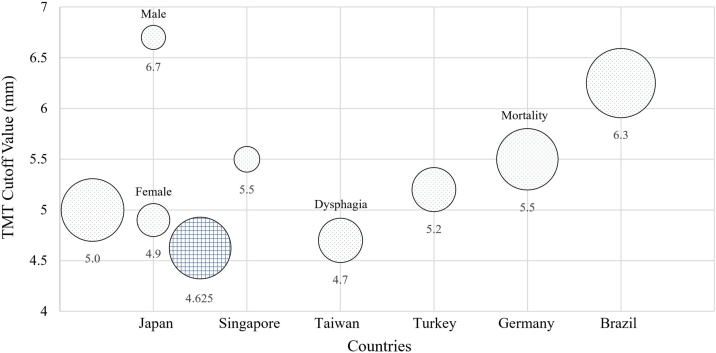
Cross-national comparison of TMT cutoff values across eight stroke studies. The diameter of each circle represents the sample size of the study in question. Dotted circles indicate studies with statistically significant results (*p* ≤ 0.05), while the grid-patterned circle represents a study with nonsignificant results (*p* > 0.05). Abbreviations: TMT = temporalis muscle thickness.

Analysis of TMT and TMA cutoff values across countries revealed significant variations. From 1708 patients across eight studies in Asia, Europe, and Latin America (Fig. 2), the findings revealed differences based on sex, ethnicity, and region. Comparative matrix synthesis of TMT cutoff values included 1060 Asian patients (Japan, Singapore, Taiwan, and Turkey), 287 Caucasian patients (Germany), and 361 Latin American patients (Brazil) [9,[37], [38], [39],^41^,^45^,^47^,^48^]. In the visual representation of the relative sample sizes, the area of each circle corresponds to the number of patients in the study in question, with the smallest circle, which represents a sample size of 45 patients, located directly above “Japan” on the x-axis and labeled “Male.” The matrix predominantly displays TMT cutoff values for combined sex populations, with circles aligned vertically representing a single study. An exception is the study by Katsuki M et al. [39], conducted in Japan, which is uniquely represented by two small circles, as it provides sex-specific TMT cutoff values: 6.7 mm for males and 4.9 mm for females. Asian cutoffs were 4.63–5.5 mm, Germany 5.5 mm, and Brazil 6.3 mm, showing increasing trends from Asia to Europe to Latin America.

For outcomes, most studies used mRS with dichotomized outcomes (0–2 good, 3–6 poor). However, Dubinski et al. analyzed continuous mRS changes from baseline instead of dichotomization [37], whereas Lim et al. used different cutoffs (0–3 good, 4–6 poor) [41]. Furthermore, two studies employed entirely different outcome measures: Yang et al. [47] measured dysphagia, whereas Gürsoy et al. [48] focused on mortality. Seven of the eight included studies found statistically significant results (P ≤ 0.05), represented by dotted circles [[37], [38], [39],^41^,^45^,^47^,^48^]. Among these, one study from Taiwan focused on dysphagia [48], and one study from Germany examined mortality [37]. The remaining studies all investigated the association between TMT and functional outcomes [38,39,41,45,47]. These distinctions are clearly visualized in the figure, with different markers used to indicate the specific measured outcomes. The only study with non-significant results (P > 0.05) is represented by a grid-patterned circle, highlighting that most studies found a statistically significant association between TMT and the measured outcomes [9].

Although data on TMA cutoff values are limited, they exhibit similar trends. Katsuki et al. [39] reported a general TMA cutoff of 200 mm², with sex-specific values of 193 mm² for females and 333 mm^2^ for males. In contrast, Rodrigues et al. [45] found a higher cutoff of 266.1 mm². These findings indicate that TMA cutoff values vary by region and sex, similar to TMT.

### 3.4 TMT and stroke outcomes

**Fig. 3:**
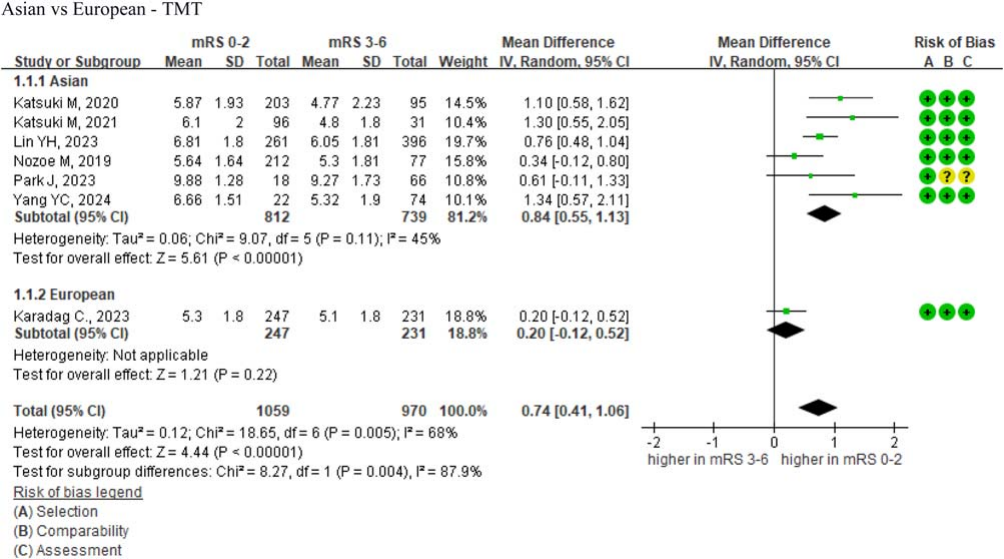
Forest plots of TMT as a predictor of functional outcome in stroke patients, stratified by ethnicity. Abbreviations: TMT = temporalis muscle thickness; mRS = modified Rankin Scale; IV random = inverse variance random-effects model; CI = confidence interval.

Our meta-analysis included seven studies that provided TMT data, encompassing 2029 patients based on the study cohorts [9,25,38,39,42,44,48]. Most studies used CT to measure TMT and defined poor outcomes as mRS 3–6 [9,25,38,39,42,48]. However, one study by Jisoo et al. utilized MRI for TMT measurements and defined a good outcome as K-MBI ≥75 [44,50]. In our pooled analysis, patients with better functional outcomes had significantly higher TMT values compared to those with poorer outcomes [9,25,38,39,42,44,48]. Based on a common TMT range of 4.8–9.88 mm, the mean TMT in the good outcome group was 0.74 mm greater than in the poor outcome group (MD = 0.74 mm, 95% CI = 0.41–1.06, *I^2^* = 68%, Fig. 3).

Because of the high heterogeneity (*I^2^* = 68%), we conducted subgroup analyses to explore potential sources. We examined factors including ethnicity, stroke type (ischemic stroke vs. hemorrhagic stroke), and the measurement tool employed (CT vs. MRI). Notably, stroke type and measurement tool analyses did not yield statistically significant differences (*P* =  0.78 and *P* =  0.72, respectively; Fig. S1). In addition, exploratory subgroup analyses based on age (<50, 50–64, 65–79, and ≥80 years) and sex (female vs. male) were conducted using subsets of studies that reported the relevant data.

Based on seven studies, the ethnicity-based subgroup analysis revealed a statistically significant difference (*P* =  0.004) [9,25,38,39,42,44,48]. In Asian populations, an increase in TMT was a statistically significant predictor of better poststroke functional outcomes (MD = 0.84 mm, 95% CI = 0.55–1.13, *I^2^* = 45%). However, in European populations, the association between the TMT and functional outcomes did not attain statistical significance (MD = 0.20 mm, 95% CI = −0.12 to 0.52). Only one eligible European study was included in the meta-analysis, precluding heterogeneity estimation for this subgroup [25].

**Fig. 4:**
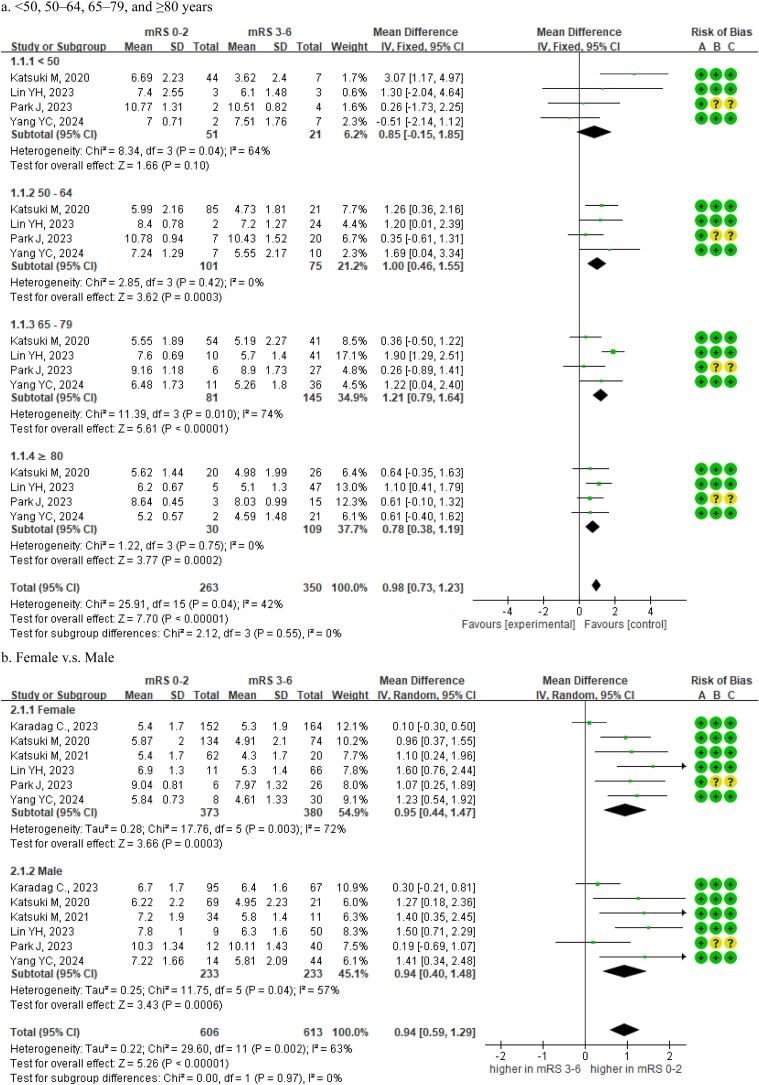
Forest plots of TMT as a predictor of functional outcome in stroke patients, stratified by (a) age and (b) sex. Abbreviations: TMT = temporalis muscle thickness; mRS = modified Rankin Scale; IV random = inverse variance random-effects model; CI = confidence interval.

Based on four studies, age-based subgroup analyses revealed distinct associations between TMT and functional outcomes [38,42,44,48]. Patients aged <50 years showed no significant association between TMT and functional outcome (MD = 0.85 mm, 95% CI: –0.15 to 1.85, *I²* = 64%). In contrast, significant associations were observed for patients aged 50–64 years (MD = 1.00 mm, 95% CI: 0.46–1.55, *I²* = 0%) and particularly in patients aged 65–79 years (MD = 1.21 mm, 95% CI: 0.79–1.64, *I²* = 74%) as well as those aged ≥80 years (MD = 0.78 mm, 95% CI: 0.38–1.19, *I²* = 0%). Substantial heterogeneity was noted in the 65–79 age subgroup, likely indicating biological or clinical variability. The test for subgroup differences was not statistically significant (*P* =  0.55, Fig. 4a), indicating that there is no evidence to conclude that age modifies the prognostic relevance of TMT.

Based on six studies, the sex-based subgroup analysis showed similar patterns [25,38,39,42,44,48]. TMT was predictive in both females (MD = 0.95 mm, 95% CI: 0.44–1.47, *I²* = 72%) and males (MD = 0.94 mm, 95% CI: 0.40–1.48, *I²* = 57%), with no significant difference in effect size (*P* =  0.97, Fig. 4b).

A GRADE assessment rated the overall quality of evidence as moderate. The subgroup analyses revealed significant demographic and ethnic variations as the primary source of heterogeneity, substantially reducing it in Asian populations (*I^2^* = 45%). Despite some imprecision, more than half of the studies showed statistical significance (*P* <  0.05) with reasonably narrow pooled CIs. Additionally, a clear dose–response gradient between TMT and functional outcomes was observed, supporting an evidence upgrade.

### 3.5 TMA and stroke outcomes

**Fig. 5:**
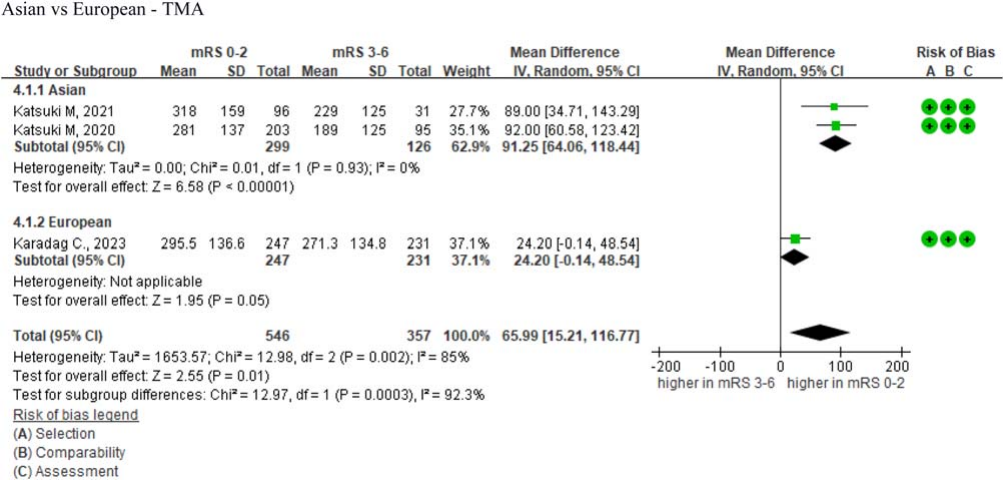
Forest plots of TMA as a predictor of functional outcome in stroke patients, stratified by ethnicity. Abbreviations: TMT = temporalis muscle thickness; IV random = inverse variance random-effects model; CI = confidence interval.

Regarding the TMA assessment, we analyzed three studies that included 903 patients, the data of which could be extracted [25,38,39]. Two studies conducted by the same researcher in Asia reported that patients with better poststroke functional outcomes had significantly higher TMA values [38,39]. The third study, which was conducted in Europe, revealed no statistical significance [25]. Our meta-analysis of these three articles revealed that higher levels of TMA were associated with better poststroke functional outcomes. According to the common TMA range of 189–318 mm^2^, patients with better functional outcomes had a mean TMA that was 65.99 mm² higher than those with poorer outcomes. (MD = 65.99 mm^2^, 95% CI = 15.21–116.77, *I^2^* = 85%, Fig. 5).

Due to the high heterogeneity (*I^2^* = 85%), we conducted subgroup analyses to explore potential sources. The subgroup analysis by ethnicity revealed significant differences (*P* =  0.003). In Asian populations, an increase in TMA was a statistically significant predictor of better poststroke functional outcomes (MD = 91.25 mm^2^, 95% CI = 64.06–118.44, *I*^2^ = 0%) [38,39]. However, in the European population, the association between TMA and functional outcomes was not statistically significant (MD = 24.20 mm^2^, 95% CI = −0.14–48.54). European data were derived from a single study; hence, no *I*^2^ value was calculated for this subgroup [25].

A GRADE assessment rated the overall quality of evidence for TMA and stroke outcomes as moderate. While the initial heterogeneity was very high (*I*^2^ = 92.3%), subgroup analyses based on ethnic differences completely eliminated this heterogeneity in Asian populations (*I*^2^ = 0%). All studies but one showed statistical significance (*P* <  0.05), with the pooled estimate demonstrating a narrow CI. Similar to TMT, a dose–response relationship was observed between TMA and functional outcomes, supporting an evidence upgrade.

### 3.6 TMT and poststroke dysphagia

**Fig. 6:**
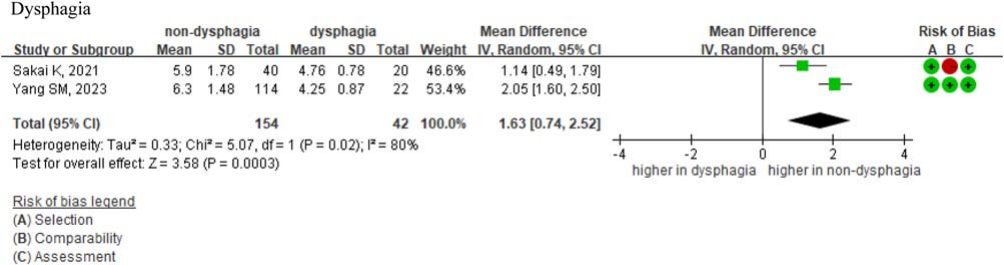
Forest plots of the TMT as a predictor of dysphagia in poststroke patients. Abbreviations: TMT = temporalis muscle thickness; IV random = inverse variance random-effects model; CI = confidence interval.

The association between the TMT and poststroke dysphagia was evaluated in two studies including a total of 196 patients [46,48]. Both studies reported that patients with poststroke dysphagia had significantly lower TMT values compared to those without dysphagia. The meta-analysis showed that, on average, patients with poststroke dysphagia had TMT values that were 1.63 mm lower than those without dysphagia (MD = 1.63 mm, 95% CI = 0.74–2.52, *I*^2^ = 50%; Fig. 6). The moderate heterogeneity (*I*^2^ = 50%) suggests some variability between the studies, which were conducted in Taiwan and Japan, possibly due to differences in patient characteristics or assessment methods. Due to the limited number of studies, we were unable to conduct sensitivity analyses to explore sources of heterogeneity.

For the association between the TMT and poststroke dysphagia, our GRADE assessment yielded low-quality evidence. The availability of only two studies prevented subgroup analyses, and the high heterogeneity (*I*^2^ = 80%) led to downgrading for inconsistency. However, a dose–response gradient was observed, supporting an evidence upgrade. The limitations in the study comprehensive number also precluded an assessment of imprecision and publication bias. Even though we have balanced these factors, the quality of evidence remained low, emphasizing the need for further research.

## 4 Discussion

Our meta-analysis is the first to report significant subgroup-specific associations of TMT with functional outcome across age, sex, and ethnicity, with the most consistent effects observed in individuals aged 50 years or older and in Asian populations. Specifically, TMT was associated with functional outcome and dysphagia, while TMA was associated with functional outcome. The overall certainty of evidence, assessed using the GRADE approach, was low to moderate. Given that TMT and TMA can be easily measured from routine cranial imaging, they represent accessible and practical tools for prognostic assessment in stroke care.

While numerous studies have established the association between sarcopenia and poor poststroke outcomes, many have traditionally relied on L3-level muscle mass measurements [51,52]. In contrast, our meta-analysis suggests that TMT and TMA may offer comparable reliability to these traditional methods, with the added advantage of accessibility due to the routine use of cranial imaging modalities in stroke care. Furthermore, Mitsiopoulos et al. support the validity of using CT or MRI for muscle mass quantification, finding no significant heterogeneity between measurements obtained via these modalities [53]. This consistency across imaging techniques further reinforces the reliability of these two parameters as measures of temporalis muscle mass and, by extension, their utility as predictors of poststroke functional outcomes.

Our study revealed notable differences in muscle thickness across various countries and ethnicities. These findings align with those of Abe et al., according to which the muscle mass of Japanese subjects was lower than that of their German and Brazilian counterparts [54]. Similarly, a study comparing Japanese and Brazilian-Japanese adolescents found more significant gains in fat-free mass and losses in body fat during maturation among Caucasian subjects, suggesting the crucial role of ethnicity in muscle development [55]. However, these variations may not be solely attributable to genetic factors. Dietary habits, especially those associated with specific regions, may play a significant role in muscle mass preservation. For instance, adherence to a Mediterranean diet has been associated with a lower incidence of sarcopenia [56]. This suggests that long-term dietary patterns could contribute to the observed differences in muscle mass across populations. These findings indicate that targeted nutritional interventions could be considered as part of comprehensive stroke rehabilitation strategies, particularly in populations predisposed to sarcopenia.

While age-related muscle loss is widely recognized, the specific impact of aging on TMT remains unclear [57]. Ian Janssen et al. reported that muscle loss rates vary by muscle group, with upper limb muscles deteriorating more slowly than lower limb ones [58]. This variability suggests that the temporalis muscle may follow a distinct age-related trajectory due to its unique anatomical role and function. In our age-based subgroup analysis, the prognostic value of TMT was apparent in individuals aged 50 years and older—including those aged 50–64, 65–79, and ≥80 years—while no significant association was observed in those under 50 years. The test for subgroup differences did not reach statistical significance, indicating that the association between TMT and functional outcome was not significantly modified by age. The observed pattern is biologically plausible and aligns with established links between aging, sarcopenia, and stroke recovery. Previous meta-analyses have confirmed the global burden of sarcopenia in older adults [59,60]. The age-related pattern observed in our study may reflect known associations between aging, muscle loss, and stroke recovery. These findings support the use of TMT as a practical prognostic marker in this vulnerable population.

Although males and females differ in absolute TMT values, our meta-analysis found no significant difference in its prognostic association with stroke outcomes across sexes. This suggests that TMT is a valid predictor in both groups, though sex-specific cutoff values may still be required for clinical use. This biological plausibility is supported by prior research, including Katsuki et al., who reported sex-influenced thresholds [39], and Abe et al., who identified comparable muscle mass differences across Japanese, Brazilian, and German populations [54]. Furthermore, the IRIDE Cohort Study in Japan reported age- and sex-specific associations between sarcopenia severity and cognitive decline in both men and women [61]. In contrast, a Chinese community-based study found that the sarcopenia index was significantly associated with sarcopenia only in males [62]. These divergent findings suggest possible sex-specific biological mechanisms. Despite these nuances, the consistent effect direction across sexes in our analysis supports the general applicability of TMT as a clinically relevant marker for prognostic evaluation in stroke patients.

Dysphagia risk in stroke patients is significantly correlated with TMT, as confirmed by this analysis synthesizing evidence from Sakai et al. [46] and Yang et al. [48]. Current techniques for the prediction of poststroke dysphagia include clinical assessments such as the water swallow test, neurological examinations such as the NIHSS, and instrumental evaluations such as videofluoroscopic swallowing studies (VFSS) or fiberoptic endoscopic evaluation of swallowing (FEES) [63,64]. While effective, these methods often require specialized equipment or expertise, potentially limiting their widespread application in acute settings. TMT estimation utilizing existing neuroimaging data from routine stroke management may offer a complementary approach. This method provides a quantifiable metric that could potentially be integrated into clinical decision-making algorithms, subject to further validation. Currently, prospective studies have validated that TMT measurements could inform personalized treatment strategies. For instance, patients categorized as high-risk based on TMT may benefit from early nutritional support and swallowing rehabilitation, aligning with guidelines emphasizing early intervention in poststroke care [65,66].

Nevertheless, this study has several limitations. First, our meta-analysis exclusively included observational studies, and we did not have data from randomized controlled trials, which may have weakened our conclusions. Second, although some included studies reported adjusted odds ratios (aORs), the definitions and covariate adjustments varied considerably, limiting comparability. To ensure methodological consistency across studies, we chose to analyze unadjusted continuous measures such as mean and standard deviation. This decision may have affected the precision of our findings and contributed to the overall quality of evidence in our GRADE assessment. Third, despite our efforts to contact all authors of the included studies for unpublished data and the successful obtention of additional data from the authors of two studies, a significant amount of extractable and analyzable data for many outcomes of interest remained unavailable. In particular, only one eligible European study was available, which may have introduced regional bias in the subgroup analysis. Forth, although we performed subgroup analyses based on age, sex, stroke subtype, ethnicity, and imaging modality, residual confounding from other unmeasured factors—such as comorbidities, nutritional status, or poststroke management—may still exist and could influence the observed associations. Finally, our measurements are intended to reflect baseline muscle status, as most TMT and TMA values were obtained early after stroke onset, before clinically significant muscle wasting would be expected. However, potential transient effects of acute-phase inflammation or cerebral edema on muscle morphology cannot be entirely ruled out. Such physiological changes during the acute phase may introduce subtle variability in early measurements [67].

## 5 Conclusion

Our meta-analysis demonstrates that TMT may serve as a useful prognostic marker in patients with stroke, particularly in individuals aged 50 years or older and in Asian populations. TMT and TMA both demonstrated prognostic value for functional outcomes, and TMT was additionally associated with dysphagia. Future research should focus on well-designed studies to establish population-specific cutoff values and validate the predictive power of TMT and TMA across diverse patient groups.

## Ethics approval

For this type of purely retrospective study, formal consent is not required.

## Declaration of Generative AI and AI-assisted technologies in the writing process

The authors declare that no generative AI or AI-assisted technologies were used in the writing process of this manuscript.

## Funding

This study was supported by 10.13039/501100011913Kaohsiung Veterans General Hospital (grant no.: KSVNSU-114-012).

## CRediT authorship contribution statement

**Yao-Chung Yang:** Writing - original draft, Investigation, Formal analysis, Data curation, Conceptualization. **Chun-Hao Yin:** Investigation, Data curation. **Pei-Chin Lin:** Writing - review & editing, Supervision, Methodology, Formal analysis, Data curation. **Yow-Ling Shiue:** Writing - review & editing, Supervision, Methodology, Formal analysis, Data curation.

## Declaration of competing interest

The authors have no relevant financial or non-financial interests to disclose.
