# Supplementary figures and images for: Association between temporalis muscle thickness and functional outcomes in acute stroke: A meta-analysis and GRADE approach
Source: J Nutr Health Aging. 2025 Jun 21;29(8):100614. doi: 10.1016/j.jnha.2025.100614 (PMC12402368; doi:10.1016/j.jnha.2025.100614)

A. ischemic stroke vs hemorrhagic stroke

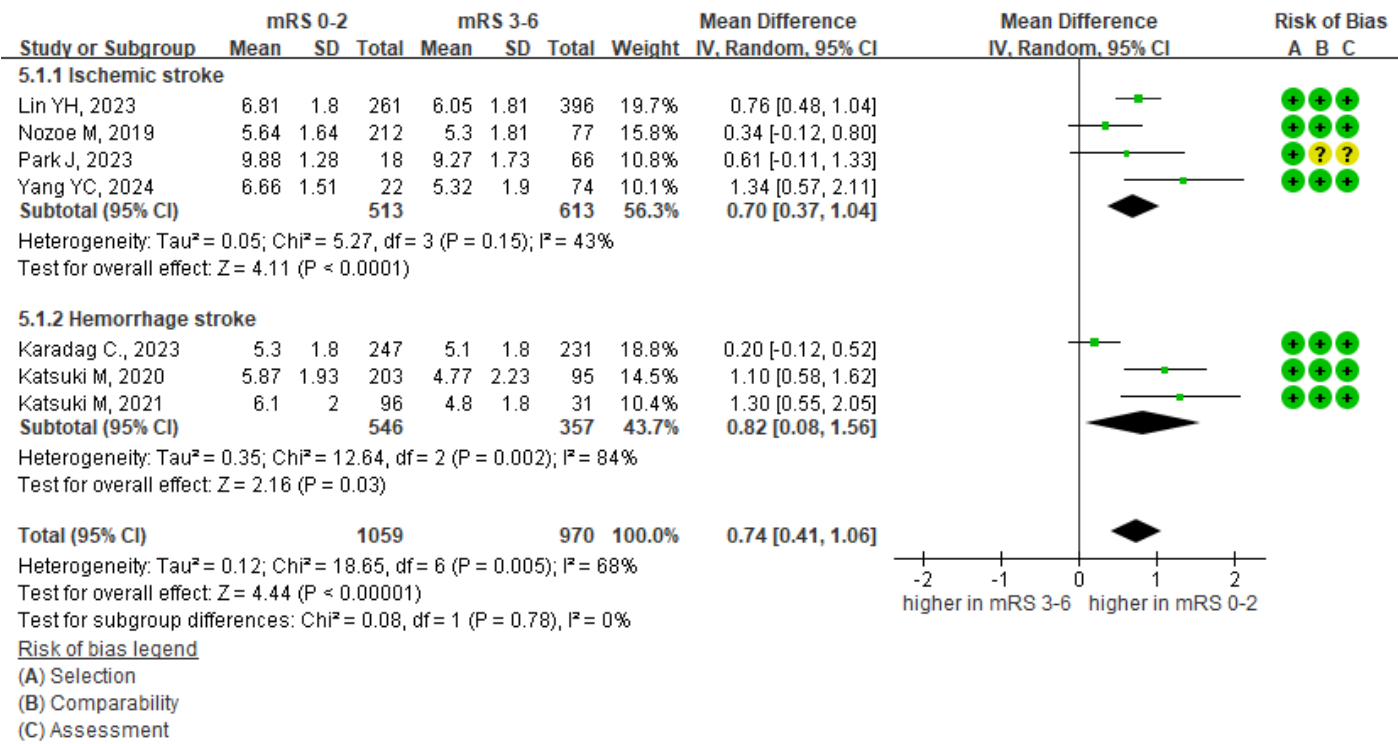

B. Computed Tomography vs Magnetic Resonance Imaging

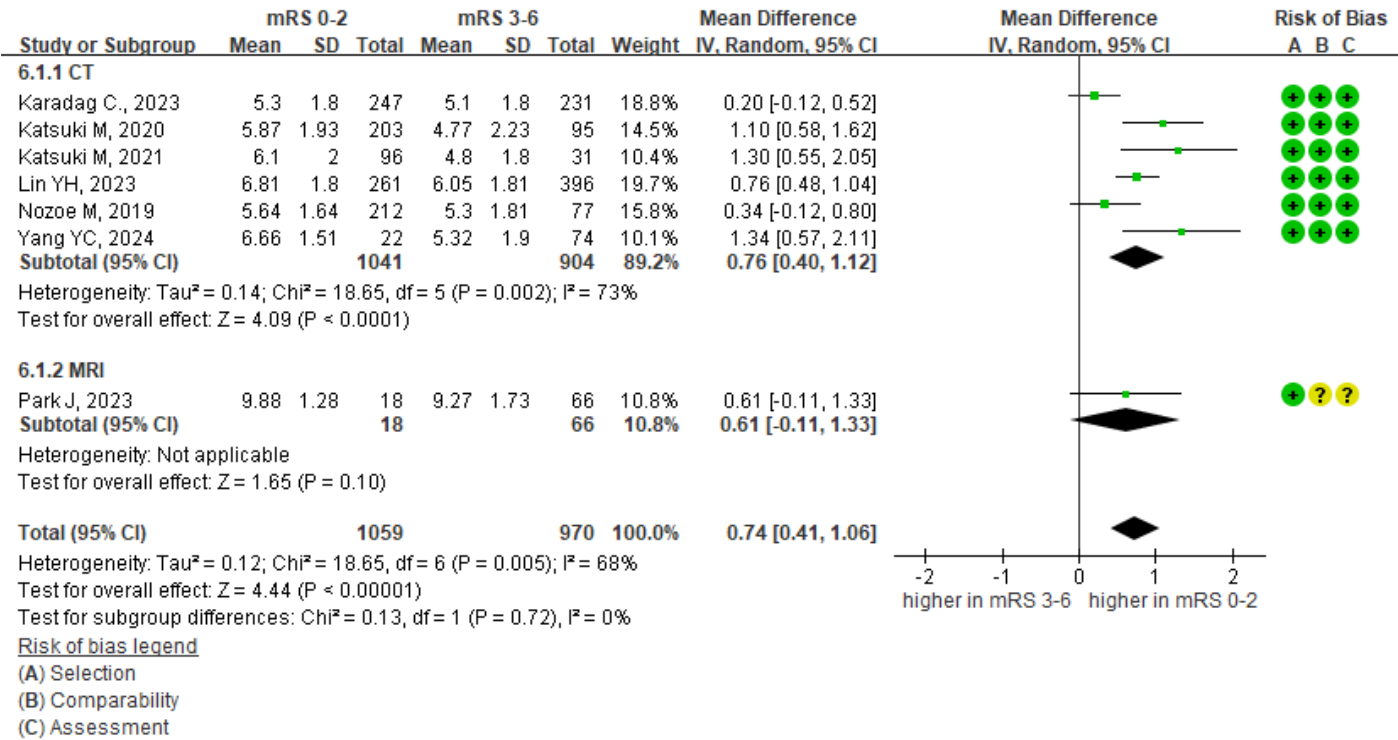

Supplement: Supplementary file 1 [file mmc1.pdf]
