# Supplementary material for: Association between temporalis muscle thickness and functional outcomes in acute stroke: A meta-analysis and GRADE approach
Source: J Nutr Health Aging. 2025 Jun 21;29(8):100614. doi: 10.1016/j.jnha.2025.100614 (PMC12402368; doi:10.1016/j.jnha.2025.100614)
Supplement: Supplementary file 2 [file mmc2.docx]

| Supplementary Table 1. Searching strategy |
| --- |
| ("intracranial haemorrhage"[All Fields] OR "intracranial hemorrhages"[MeSH Terms] OR ("intracranial"[All Fields] AND "hemorrhages"[All Fields]) OR  "intracranial hemorrhages"[All Fields] OR ("intracranial"[All Fields] AND "hemorrhage"[All Fields]) OR "intracranial hemorrhage"[All Fields] OR  ("subarachnoid haemorrhage"[All Fields] OR "subarachnoid hemorrhage"[MeSH Terms] OR ("subarachnoid"[All Fields] AND "hemorrhage"[All Fields]) OR  "subarachnoid hemorrhage"[All Fields]) OR ("intracerebral haemorrhage"[All Fields] OR "cerebral hemorrhage"[MeSH Terms] OR ("cerebral"[All Fields] AND "hemorrhage"[All Fields]) OR  "cerebral hemorrhage"[All Fields] OR ("intracerebral"[All Fields] AND "hemorrhage"[All Fields]) OR "intracerebral hemorrhage"[All Fields]) OR ("stroke"[MeSH Terms] OR  "stroke"[All Fields] OR "strokes"[All Fields] OR "stroke s"[All Fields])) AND  ("temporal muscle"[MeSH Terms] OR ("temporal"[All Fields] AND "muscle"[All Fields]) OR  "temporal muscle"[All Fields] OR ("temporalis"[All Fields] AND "muscle"[All Fields]) OR "temporalis muscle"[All Fields] OR  ("temporal muscle"[MeSH Terms] OR ("temporal"[All Fields] AND "muscle"[All Fields]) OR  "temporal muscle"[All Fields])) |
