# Supplementary material for: Association between temporalis muscle thickness and functional outcomes in acute stroke: A meta-analysis and GRADE approach
Source: J Nutr Health Aging. 2025 Jun 21;29(8):100614. doi: 10.1016/j.jnha.2025.100614 (PMC12402368; doi:10.1016/j.jnha.2025.100614)
Supplement: Supplementary file 3 [file mmc3.docx]

| Supplementary Table 2. Risk of bias assessment for cohort studies using the Newcastle–Ottawa Scale | | | | | | | | | | |
| --- | --- | --- | --- | --- | --- | --- | --- | --- | --- | --- |
| **Study** | **Risk of bias assessment** | | | | | | | | | |
|  | **Selection** | | | | **Comparability** | | **Outcome / Exposure** | | | **Total score** |
| Dubinski D, 2023 | * | * | * | * | * | * | * | * | * | 9 |
| Karadag C, 2023 | * | * | * | * | * | * | * | * | * | 9 |
| Katsuki M, 2020 | * | * | * | * | * | * | * | * | * | 9 |
| Katsuki M, 2021 | * | * | * | * | * | * | * | * | * | 9 |
| Li YX, 2022 | * | * | * | * | * | * | * |  | * | 8 |
| Lim JX, 2022 | * | * | * | * | * |  | * | * | * | 8 |
| Lin YH, 2023 | * | * | * | * | * | * | * | * | * | 9 |
| Namgung HG, 2023 | * | * | * | * | * | * | * | * | * | 9 |
| Nozoe M, 2019 | * | * | * | * | * | * | * | * | * | 9 |
| Park J, 2023 | * | * | * | * | * |  | * |  | * | 7 |
| Rodrigues RS, 2022 | * | * | * | * | * | * | * | * | * | 9 |
| Sakai K, 2021 | * | * | * | * |  |  | * | * | * | 7 |
| Tutal Gürsoy G, 2023 | * | * | * | * | * | * | * | * | * | 9 |
| Yang SM, 2023 | * | * | * | * | * | * | * | * | * | 9 |
| Yang YC, 2024 | * | * | * | * | * | * | * | * | * | 9 |
